# Computational modelling of pathogenic protein spread in neurodegenerative diseases

**DOI:** 10.1371/journal.pone.0192518

**Published:** 2018-02-05

**Authors:** Konstantinos Georgiadis, Selina Wray, Sébastien Ourselin, Jason D. Warren, Marc Modat

**Affiliations:** 1 Translational Imaging Group, Centre for Medical Image Computing, Department of Medical Physics and Biomedical Engineering, University College London, London NW1 2HE, United Kingdom; 2 Department of Molecular Neuroscience, Institute of Neurology, University College London, London WC1N 3BG, United Kingdom; 3 Dementia Research Centre, Institute of Neurology, University College London, London WC1N 3BG, United Kingdom; McGill University, CANADA

## Abstract

Pathogenic protein accumulation and spread are fundamental principles of neurodegenerative diseases and ultimately account for the atrophy patterns that distinguish these diseases clinically. However, the biological mechanisms that link pathogenic proteins to specific neural network damage patterns have not been defined. We developed computational models for mechanisms of pathogenic protein accumulation, spread and toxic effects in an artificial neural network of cortical columns. By varying simulation parameters we assessed the effects of modelled mechanisms on network breakdown patterns. Our findings suggest that patterns of network breakdown and the convergence of patterns follow rules determined by particular protein parameters. These rules can account for empirical data on pathogenic protein spread in neural networks. This work provides a basis for understanding the effects of pathogenic proteins on neural circuits and predicting progression of neurodegeneration.

## Introduction

Accumulation of pathogenic protein in neural tissue is the core process underpinning neurodegenerative brain pathologies and ultimately responsible for their phenotypic consequences. An emerging paradigm of neurodegeneration emphasises the propagation of pathogenic proteins across neural networks, leading to consistent spatiotemporal profiles of regional brain dysfunction and atrophy that can be mapped macroscopically using neuroimaging techniques [1–4]. Certain features of pathogenic proteins such as conformational misfolding and the propensity to ‘template’ the conversion of normal protein to pathogenic form favour the spread of proteinopathies [5] while in vitro seeding and animal inoculation studies suggest that protein spread co-opts neural circuitry [6, 7]. It has been proposed that neurodegenerative phenotypes are the result of specific conjunctions of pathogenic protein and neural circuit characteristics: ‘molecular nexopathies’ [3]. However, the mechanisms that link protein accumulation to neural network breakdown are still poorly understood. Elucidating these mechanisms would transform the diagnosis and tracking of neurodegenerative diseases and inform the design of rational disease-modifying therapies.

Human neuroimaging techniques are remote from the local tissue effects that induce neurodegeneration while in vitro and in vivo systems are resource-and time-intensive. Computational approaches would potentially allow rapid evaluation of neurodegeneration models and derivation of relevant parameters of protein accumulation and spread. Most computational research on these diseases has focused on classification and prediction of atrophy [2, 8] rather than the elucidation of underlying mechanisms. However, computational modelling approaches are potentially of much wider utility, as illustrated by previous work applying such methods to study the aggregation of amyloid-beta and tau in Alzheimer’s disease and evaluate therapeutic interventions [9].

Here we describe a computational modelling approach to simulate mechanisms of pathogenic protein accumulation, spread and toxic effects within an artificial small neural network. Using the NEURON simulator software [10], we simulated an artificial neural network comprised of cortical columns [11], a representative and frequent target of neurodegenerative diseases [12]. This network has been previously used to simulate pathological neuronal communication in Parkinson’s disease and Alzheimer’s disease [13, 14]. We addressed the general hypothesis that this model would generate protein and network dependent disease effects, in line with empirical data for protein spread and macroscopic disease behavior. The molecular nexopathies paradigm predicts that structural features of neural circuits confer vulnerability to particular pathogenic proteins [3]. To test this hypothesis, we ran simulations, systematically varying protein and network parameters and we defined metrics that relate these parameter variations to protein spread and the network damage pattern.

All computational models entail simplifying assumptions. For example, pathogenic proteins often possess a number of conformational isoforms [15], but we reduced this variation to model a normally folded and a pathogenically misfolded variant. We modelled protein solubility and misfolding properties, shown in vivo to be key determinants of cell integrity and survival [15]. In addition, we modelled protein spread through passive diffusion, active transport and synaptic transfer, all of which are characteristics relevant to network spread [3, 4, 6, 7]. Identification of disease-specific network signatures is challenging in the presence of stochastic variation (observed for example, between brain atrophy profiles of individual patients). Here we used time to convergence of simulations to assess how robustly and consistently protein and network parameters contribute to establishing patterns of spread. The null hypothesis (no effect of modifying protein and network parameters on spread) would predict no convergence between simulations. We also assessed how these parameters affect neural network survival and asymmetry of network damage (key features of protein spread in real neural networks [3, 6]).

## Materials and methods

**Fig 1:**
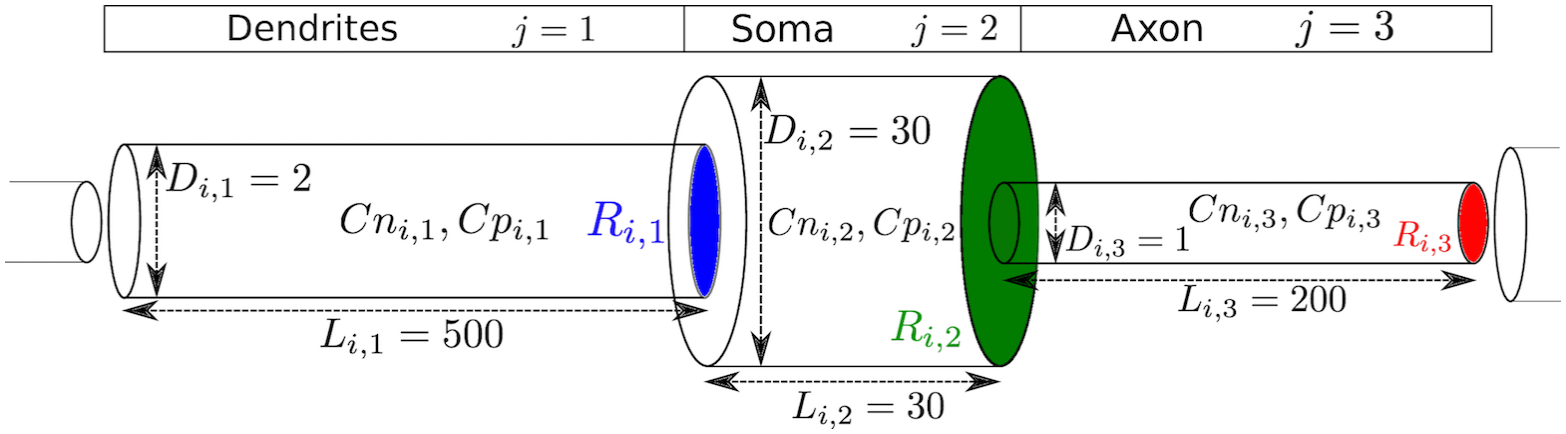
A visual representation of a simulated neuron and its section parameters.

We used NEURON, a simulator for neural networks [10] and focused our simulations on the interaction between pathogenic protein and cortical columns [12], based on the neural network used by Neymotin *et al.* [11]. This network had *K* = 3 cortical columns, each with 470 neurons (*N* = 1410 total). Each neuron *i* ∈ {1, …, *N*}, belongs to a cortical column *Col*(*i*) = {1, 2, 3}, to a layer *Lay*(*i*) = {2, 4, 5, 6} and has a type which can be excitatory Regular Spiking (RS), excitatory Intrinsically Bursting (IB), inhibitory Fast Spiking (FS) or inhibitory Low-Threshold Spiking (LTS), *Type*(*i*) = {*RS*, *IB*, *FS*, *LTS*}. Each neuron is modelled with 3 cylindrical elements, called sections: one for its dendrites (*j* = 1), one for its soma (*j* = 2) and one for the axon (*j* = 3). A concentration of non-pathogenic (normal) protein $Cn_{i,j}^{t}$ and pathogenic (misfolded) protein $Cp_{i,j}^{t}$ exists within each section at time *t*. We set *C*_*max*_ = 1 as the maximum concentration, *i.e.* $Cn_{i,j}^{t}+Cp_{i,j}^{t}\leq C_{max}$. Many neuronal properties and the neural network’s connectivity have been defined by Neymotin *et al.* [11] and for our purposes we defined the diameter *D*_*i*,*j*_, length *L*_*i*,*j*_, base area *R*_*i*,*j*_ = *π*(*D*_*i*,*j*_/2)^2^ and volume *V*_*i*,*j*_ = *L*_*i*,*j*_ × *R*_*i*,*j*_ of each section, as well as $r_{i,j\to \bar{i},\bar{j}}$ as the strength of a synapse starting from neuron *i*, connecting to the postsynaptic neuron $\bar{i}$, section $\bar{j}$. If there is no synaptic connection, then $r_{i,j\to \bar{i},\bar{j}}=0$. We defined $p_{i,j\to \bar{i},\bar{j}}$ as the intrinsic tendency of protein to spread selectively via intercolumnar synaptic connections (the projection neurons for each column), which drive protein spread across the network. Fig 1 is a schematic representation of a simulated neuron.

We modelled protein production, misfolding, clearance, passive diffusion, active transport, synaptic transfer, a toxic effect on the firing frequencies of neurons, neuronal toxicity (overall damage to the neuron) and neuronal death. These processes were applied for all neurons, at every timestep (simulation iteration), in the aforementioned order and we use the notation $Cn_{i,j}^{t,\tau }$ and $Cp_{i,j}^{t,\tau }$ to signify intermediate updates to the concentrations, where *τ* indicates an intermediate timestep. Except for misfolding, these processes were applied in the same manner for both normal and pathogenic proteins. Therefore, henceforth we will only refer to pathogenic protein (readers can assume similar equations for normal protein).

### Simulation setup

Common settings for all simulations were: timestep interval *dt* = 0.025msec, passive diffusion fraction *f*_*pd*_ = 0.05, production and clearance rates *R*_*Pn*_ = *R*_*Cn*_ = 0.0002, *R*_*Pp*_ = *R*_*Cp*_ = 0.00002 and normal concentration levels *C*_*nn*_ = *C*_*pn*_ = 0.01. We ran 11016 simulations, varying eight parameters: 1) two random instances of neural network connectivities based on the connection densities in Table 2 of Lytton *et al.* [11]; 2) soluble/clearable (there is evidence suggesting soluble tau and amyloid-beta oligomers are toxic, whereas larger, insoluble aggregates are not toxic [16]) or insoluble/unclearable pathogenic protein (in the latter case there is no clearance and negligible production of pathogenic protein); 3) 17 types of seeds—in the first three types (Seed All 1, 2 or 3) all neurons start with $Cn_{i,j}^{t=0}=Cp_{i,j}^{t=0}=0.01$ in simulations with soluble pathogenic protein and $Cn_{i,j}^{t=0}=0.01,Cp_{i,j}^{t=0}=0.0001$ with insoluble pathogenic protein (the difference between these three seeds was variation in the random number generation of NEURON)—for the remaining types of seeds we added extra pathogenic protein at the start of the simulation to a single neuron of the first cortical column, varying the cortical layer and neuronal type of the seeded neuron (Seed L2RS); 4) low misfolding rate *R*_*M*_ = 0.08 or high misfolding rate *R*_*M*_ = 0.09; 5) no diffusion, low diffusion speed *σ*_*z*_ = 50 or high diffusion speed *σ*_*z*_ = 500; 6) no active transport *f*_*at*_ = 0, weak active transport *f*_*at*_ = 0.0001 or strong active transport *f*_*at*_ = 0.001; 7) three settings for synaptic transfer and the toxic effect of the pathogenic protein—no synaptic transfer *f*_*st*_ = 0 with an increase of voltage thresholds $V_{th\_adapt_{i}}$, low synaptic transfer *f*_*st*_ = 1 with an increase of voltage thresholds or high synaptic transfer *f*_*s*_ *t* = 1 with a decrease of voltage thresholds (since a decrease of voltage thresholds increases firing frequencies, this indirectly strengthens the synaptic transfer mechanism); 8) tendency to avoid intercolumnar connections $p_{i,j\to \bar{i},\bar{j}}=0.01$, no spread selectivity $p_{i,j\to \bar{i},\bar{j}}=1$ or tendency to spread via intercolumnar connections $p_{i,j\to \bar{i},\bar{j}}=100$.

### Production

Pathogenic protein could in general be created by the processes either of transcription and translation (production) and/or by post-translational modification (misfolding) of normal protein. We assumed production remains at a specific rate of protein molecules per unit of time (with little variation), which is unaffected by protein accumulation [17], until cellular death. We modelled production as $an_{i,j}^{t}$, $ap_{i,j}^{t}$ (Eq 1), which were samples from normal distributions, based on mean production rates *R*_*Pn*_, *R*_*Pp*_:

$$\begin{array}{ccc} ap_{i,2}^{t} & ∼ & N(R_{Pp},{R_{Pp}}^{2}),ap_{i,1}^{t}=ap_{i,3}^{t}=0 \end{array}$$ (1)

$$\begin{array}{ccc} Cp_{i,j}^{t,1} & = & Cp_{i,j}^{t}+ap_{i,j}^{t} \end{array}$$ (2)

### Misfolding

**Fig 2:**
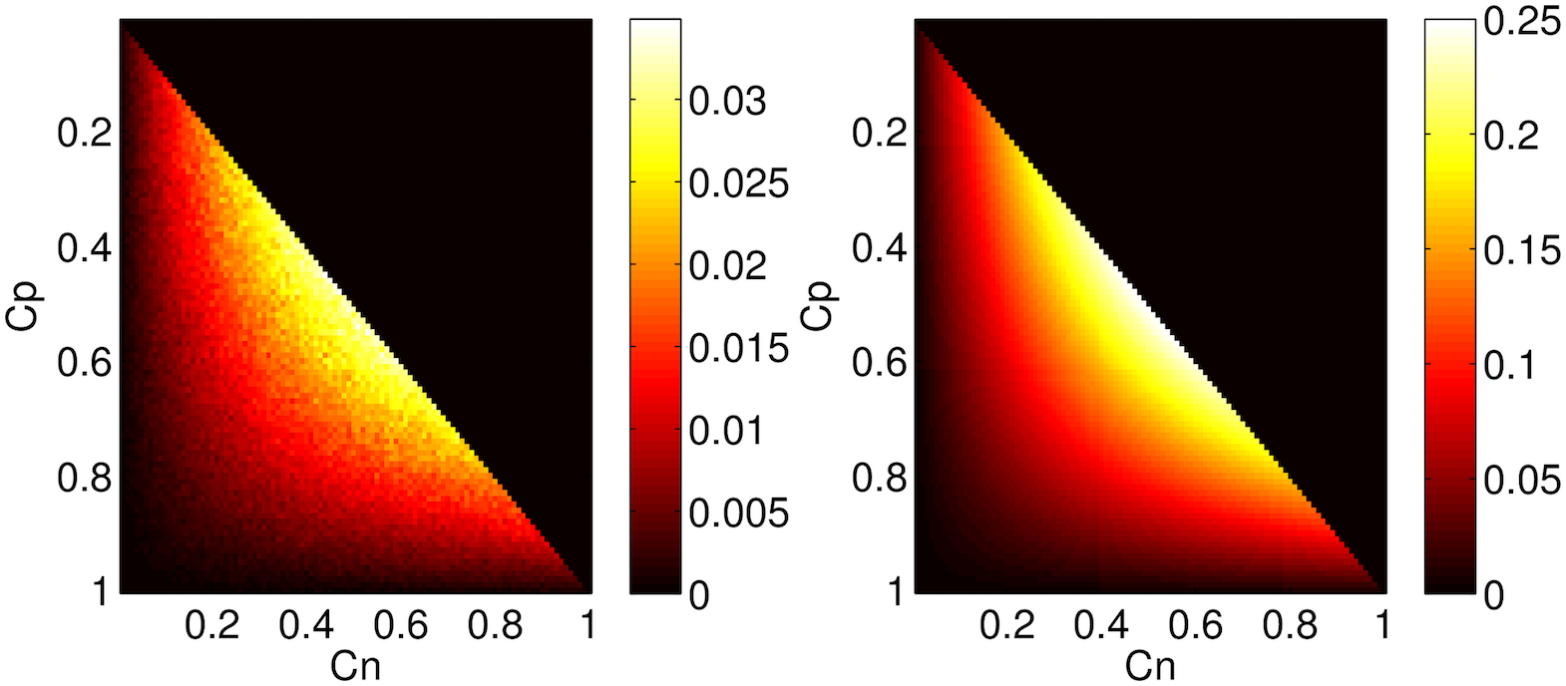
Left: Concentration of normal protein (*Cn*) misfolded to pathogenic (*Cp*) based on simulations. Right: Proposed misfolding model: *R*_*M*_ * *Cn* * *Cp*, with *R*_*M*_ = 1. Adjusting *R*_*M*_ can negate the scaling difference.

Generally, the proteins associated with neurodegeneration are misfolded from a soluble to an insoluble state, with a number of intermediates and it is primarily larger aggregates that cause normal protein to misfold [18]. We hypothesised that normal protein is misfolded when it comes in close proximity to pathogenic protein [3, 7, 15]. To derive a model based on our hypothesis, we simulated a cube volume, within which we added normal and pathogenic protein molecules and let them diffuse. Normal protein molecules would misfold and become pathogenic whenever they came in close proximity. At the end of the simulation we calculated the concentration of normal protein molecules that misfolded. We repeated this simulation varying the initial number of normal and pathogenic protein concentrations. Considering simulation results (Fig 2, left) as ground truth, our proposed model of the product of the two concentrations (Eq 3, Fig 2, right), was in close agreement with the ground truth:

$$\begin{array}{ccc} b_{i,j}^{t} & = & R_{M}Cn_{i,j}^{t}Cp_{i,j}^{t} \end{array}$$ (3)

$$\begin{array}{ccc} Cn_{i,j}^{t,2} & = & Cn_{i,j}^{t,1}-b_{i,j}^{t}\\ Cp_{i,j}^{t,2} & = & Cp_{i,j}^{t,1}+b_{i,j}^{t} \end{array}$$ (4)

### Clearance

Protein accumulation is caused by an imbalance between overall protein production and clearance due to three mutually compatible scenarios: (a) clearance mechanisms remain healthy, but insoluble pathogenic proteins accumulate, (b) clearance mechanisms remain healthy, but are overwhelmed by the accumulation of protein due to other mechanisms, or (c) clearance mechanisms weaken, reducing clearance rates [19, 20]. We modelled protein clearance such that the concentration of every protein variant tends to be maintained at a normal level within a cell. When protein levels are below or above normal, production rates remain unchanged, but clearance rates adapt to return protein levels to normality. Given clearance rates *R*_*Cn*_, *R*_*Cp*_ at normal levels of protein concentration *C*_*nn*_, *C*_*pn*_, the protein clearance terms $qn_{i,j}^{t}$, $qp_{i,j}^{t}$ (Eq 5) were:

$$\begin{array}{ccc} qp_{i,j}^{t} & ∼ & N(\mu_{Cp}^{t},{\mu_{Cp}^{t}}^{2}) \end{array}$$ (5)

$$\begin{array}{ccc} \mu_{Cp}^{t} & = & R_{Cp}\log(1+\left(e-1\right)\frac{Cp_{i,j}^{t}}{C_{pn}}) \end{array}$$ (6)

$$\begin{array}{ccc} Cp_{i,j}^{t,3} & = & Cp_{i,j}^{t,2}-qp_{i,j}^{t}, \end{array}$$ (7)

where $\mu_{Cn}^{t}$, $\mu_{Cp}^{t}$ are considered as the adaptive clearance rates. Although our choice of a logarithmic function is somewhat arbitrary, it is adaptive to the concentration levels and biologically plausible.

### Normal and pathogenic protein equilibrium

**Fig 3:**
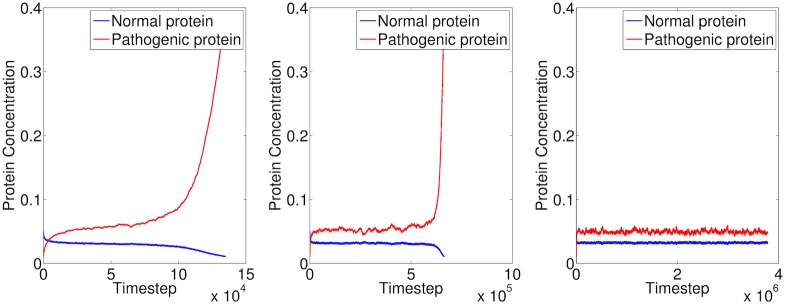
Three simulations showing the equilibrium between normal and pathogenic protein within a single volume, in conjunction with our modelled production, misfolding and clearance, with different misfolding rates *R*_*M*_. The initial protein concentrations are *Cn* = 0.05, *Cp* = 0.01, the normal concentration levels are *C*_*nn*_ = 0.05, *C*_*pn*_ = 0.01 and the production and clearance rates are *R*_*Pn*_ = *R*_*Cn*_ = 4*e* − 4, *R*_*Pp*_ = *R*_*Cp*_ = 0.8*e* − 4. Left: *R*_*M*_ = 0.0626. Middle: *R*_*M*_ = 0.0624. Right: *R*_*M*_ = 0.622. Note the different time scales.

The equilibrium between normal and pathogenic protein concentrations is commonly hypothesised to be lost in some neurodegenerative diseases [21]. We ran simulations with normal and pathogenic protein concentration at a single point, varying the misfolding rate *R*_*M*_ to observe behavioural differences on the protein equilibrium (Fig 3). When *R*_*M*_ = 0.0626, the protein concentrations reached equilibrium, but after a small number of timesteps this equilibrium was lost. When *R*_*M*_ = 0.0624, the same process occurred, but the equilibrium was lost after a larger number of timesteps. When *R*_*M*_ = 0.622, the equilibrium was maintained after a much larger number of timesteps. Notably, the behaviour of this process is sensitive to small changes to *R*_*M*_. While these were simple simulations, biochemical variations of this kind could contribute to individual variation in vulnerability to neurodegenerative diseases. Protein aggregation may occur all the time, but in most individuals, neurons may be able to degrade small soluble aggregates, preventing the formation of large insoluble aggregates [18]. Tau strains that seed more efficiently (*i.e.* have a high misfolding rate) may be significantly more toxic to cells that express high levels of monomeric tau [22]. These simulations demonstrate how these two hypotheses could be true.

### Spread

There are many hypotheses regarding the spread of pathogenic proteins [7, 15, 23] (*e.g.* diffusion, exocytosis, *etc.*). Proteins spread primarily through the neural network [16, 24], in either anterograde or retrograde fashion [24]. We modelled intracellular and intercellular passive diffusion, intracellular active transport and intercellular synaptic transfer. We defined *f*_*pd*_, *f*_*at*_ and *f*_*st*_ as fractions, restricting the maximum protein quantity that can spread out of a neuronal section within a single timestep for passive diffusion, active transport and synaptic transfer respectively. Based on available empirical data [18, 25], we modelled both normal and pathogenic protein spread via the same mechanisms.

#### Passive diffusion

We modelled passive diffusion of protein as Brownian motion, restricted to the cylindrical shape of neuronal sections intracellularly and the synapses and their synaptic strengths intercellularly. We defined $z_{i,j\to \bar{i},\bar{j}}$ (Eq 10) as the fraction of $Cp_{i,j}^{t,3}$ that diffuses past the boundary of section *i*, *j* and enters a neighbouring section $\bar{i}$, $\bar{j}$. These coefficients comprise four terms: 1) $y_{i,j\to \bar{i},\bar{j}}$ models Brownian motion in the primary spatial dimension by taking the integral of the one-dimensional normal distribution with its mean at the centre of *i*, *j* and its standard deviation *σ*_*z*_ controls the speed of passive diffusion (Eq 8); 2) the ratio of the area of the entrance to the source’s base area *R*_*i*,*j*_ accounts for the other two spatial dimensions; 3) predetermined weights $w_{i,j\to \bar{i},\bar{j}}$ had values equal to the synaptic strengths for intercellular spread or were set arbitrarily to a high value for intracellular spread; 4) the intrinsic spread selectivity of the protein $p_{i,j\to \bar{i},\bar{j}}$.

$$\begin{array}{ccc} y_{i,j\to \bar{i},\bar{j}} & = & \frac{1}{\sqrt{2\pi }}\int_{-\infty }^{\frac{-L_{i,j}}{2\sigma_{z}}}e^{\frac{x^{2}}{2}}dx,\;\text{if}\;\bar{i},\bar{j}\;\text{neighbour}\;\text{of}\;i,j \end{array}$$ (8)

$$w_{i,j\to \bar{i},\bar{j}}=\{\begin{array}{l} 20,\;\text{if}\;i=\bar{i}\\ r_{i,j\to \bar{i},\bar{j}},\;\text{if}\;i\neq \bar{i} \end{array}$$ (9)

$$\begin{array}{ccc} z_{i,j\to \bar{i},\bar{j}} & = & y_{i,j\to \bar{i},\bar{j}}\frac{\min(R_{i,j},R_{\bar{i},\bar{j}})}{R_{i,j}}w_{i,j\to \bar{i},\bar{j}}p_{i,j\to \bar{i},\bar{j}} \end{array}$$ (10)

However, this model spreads protein to neighbouring neuronal sections only and not to every geodesically nearby section. We calculated coefficients $d_{i,j\to \bar{i},\bar{j}}$, indicating what fraction of $Cp_{\bar{i},\bar{j}}^{t,3}$ spreads to section *i*, *j* using the following approach: choose a source section $\bar{i}$, $\bar{j}$ and spread fractions of $Cp_{\bar{i},\bar{j}}^{t}$ to its neighbour sections based on $z_{\bar{i},\bar{j}\to i,j}$. Then, iterate the following: if the amount of protein that a neighbour section received is above a threshold *t*_*sig*_, repeat the process for every such section as a source, spreading the quantity those sections received, until convergence to an equilibrium. The updated concentrations are (Eq 13):

$$\begin{array}{ccc} x_{k}^{t} & = & Cp_{i,j}^{t,3}V_{i,j} \end{array}$$ (11)

$$M_{k,l}=\{\begin{array}{l} f_{pd}d_{i,j\to \bar{i},\bar{j}},k\neq l\\ 1-f_{pd}+f_{pd}d_{i,j\to \bar{i},\bar{j}},k=l \end{array}$$ (12)

$$\begin{array}{ccc} Cp_{i,j}^{t,4} & = & \frac{M_{k,:}x^{t}}{V_{i,j}}, \end{array}$$ (13)

where $k=3\left(i-1\right)+j,l=3\left(\bar{i}-1\right)+\bar{j}$ and the notation **M**_*k*,:_ is the row vector consisting of the elements of the *k*-th row of **M**.

#### Active transport

We modelled active transport based on data for the axonal transport of tau protein [24, 26], a relevant pathogenic protein in many neurodegenerative diseases. We concluded that tau shows anterograde movement, no movement and retrograde movement 15.4%, 73%, 11.6% of the time, respectively. Due to lack of evidence for transport rates in dendrites and somas, we adjusted these percentages for all neuronal sections, favouring anterograde movement. We defined $e_{i,j\to \bar{i},\bar{j}}^{t}$ as the fraction of protein that is actively transported from *i*, *j* to $\bar{i}$, $\bar{j}$ at time *t*.

$$\begin{array}{c} Cp_{i,j}^{t,5}=\frac{\sum_{\bar{j}}e_{i,\bar{j}\to i,j}^{t}Cp_{i,\bar{j}}^{t,4}V_{i,\bar{j}}}{V_{i,j}} \end{array}$$ (14)

#### Synaptic transfer

We hypothesised that every action potential transfers a large quantity of protein to postsynaptic neurons. There is evidence that more tau (250% increase [25]) and amyloid-beta are released from neurons when those neurons are actively stimulated [16, 25]. We defined $g_{i,j\to \bar{i},\bar{j}}^{t}$ as the fraction of protein that is synaptically transferred when an action potential occurs.

$$\begin{array}{ccc} g_{i,j\to \bar{i},\bar{j}}^{t} & = & \frac{0.154f_{st}p_{i,j\to \bar{i},\bar{j}}r_{i,j\to \bar{i},\bar{j}}}{\sum_{\tilde{i}}\sum_{\tilde{j}}p_{i,j\to \tilde{i},\tilde{j}}r_{i,j\to \tilde{i},\tilde{j}}} \end{array}$$ (15)

$$\begin{array}{ccc} Cp_{i,j}^{t+1} & = & \frac{\sum_{\bar{i}}\sum_{\bar{j}}g_{\bar{i},\bar{j}\to i,j}^{t}Cp_{\bar{i},\bar{j}}^{t,5}V_{\bar{i},\bar{j}}}{V_{i,j}} \end{array}$$ (16)

### Neuronal toxicity, toxic effects and cellular death

We assumed that the toxicity level within neurons, $txc_{i}^{t}$ (starting at 0), increases due to protein accumulation, based on an exponential function of the protein concentration (Eq 17). There are many hypotheses regarding the toxic effects of pathogenic proteins [3, 21, 23]. Amyloid-beta may cause hyperexcitability [16, 25], whereas tau causes synaptic loss [16]. We modelled that neuronal toxicity causes a toxic effect on the voltage threshold $V_{th\_adapt_{i}}$ [11] required for triggering an action potential. As neuronal toxicity increases, the threshold either increases, indirectly reducing firing frequencies (toxic loss of function) or decreases, indirectly increasing firing frequencies (toxic gain of function). Once toxicity reached $txc_{i}^{t_{i}}=1$, we assumed cellular death occurred at time *t*_*i*_, after which only the processes of misfolding and diffusion out of the neuron would continue.

$$\begin{array}{c} txc_{i}^{t}=txc_{i}^{t-1}+0.001(\exp(10\sum_{j}\frac{Cn_{i,j}^{t}+Cp_{i,j}^{t}}{3})-1) \end{array}$$ (17)

### Results metrics

$$\begin{array}{ccc} SSG_{i} & = & \sum_{\forall \bar{i}\setminus i,\forall \bar{j},\forall j}r_{i,j\to \bar{i},\bar{j}}-\sum_{\forall \bar{i}\setminus i,\forall \bar{j},\forall j}r_{\bar{i},\bar{j}\to i,j} \end{array}$$ (18)

$$\begin{array}{ccc} D_{s,\bar{s}}\left(n\right) & = & \frac{\#\{G^{s}\left(n\right)\cap G^{\bar{s}}\left(n\right)\}}{n} \end{array}$$ (19)

$$\begin{array}{lll} t_{c}\left(s,\bar{s}\right) & = & \underset{n}{\text{arg}\;\text{min}}\left|D_{s,\bar{s}}\left(n\right)-t_{conv}\right|,\\ & & s.t.\;D_{s,\bar{s}}\left(m\right)\geq t_{conv},\forall m\in \left\{t_{c}\left(s,\bar{s}\right),\ldots ,N\right\} \end{array}$$ (20)

$$\begin{array}{ccc} {\text{CONV}}_{ij} & = & \frac{\sum_{s\in H_{i}}\sum_{\bar{s}\in H_{j}}t_{c}\left(s,\bar{s}\right)}{|H_{i}\left|\right|H_{j}|} \end{array}$$ (21)

$$\begin{array}{ccc} ASY & = & \max_{t}\sqrt{\frac{\sum_{i}{\left(txc_{i}^{t}-\sum_{j}txc_{j}^{t}/N\right)}^{2}}{N-1}} \end{array}$$ (22)

## Results

We ran 11016 simulations, varying eight parameters (see Simulation Setup under Materials and methods): network connectivity, protein seed location, pathogenic protein solubility, misfolding rate, passive diffusion speed, active transport rate, synaptic transfer and the tendency of protein to spread selectively via intercolumnar synaptic connections (predicted to be a key determinant of the pattern of circuit breakdown [3]).

**Fig 4:**
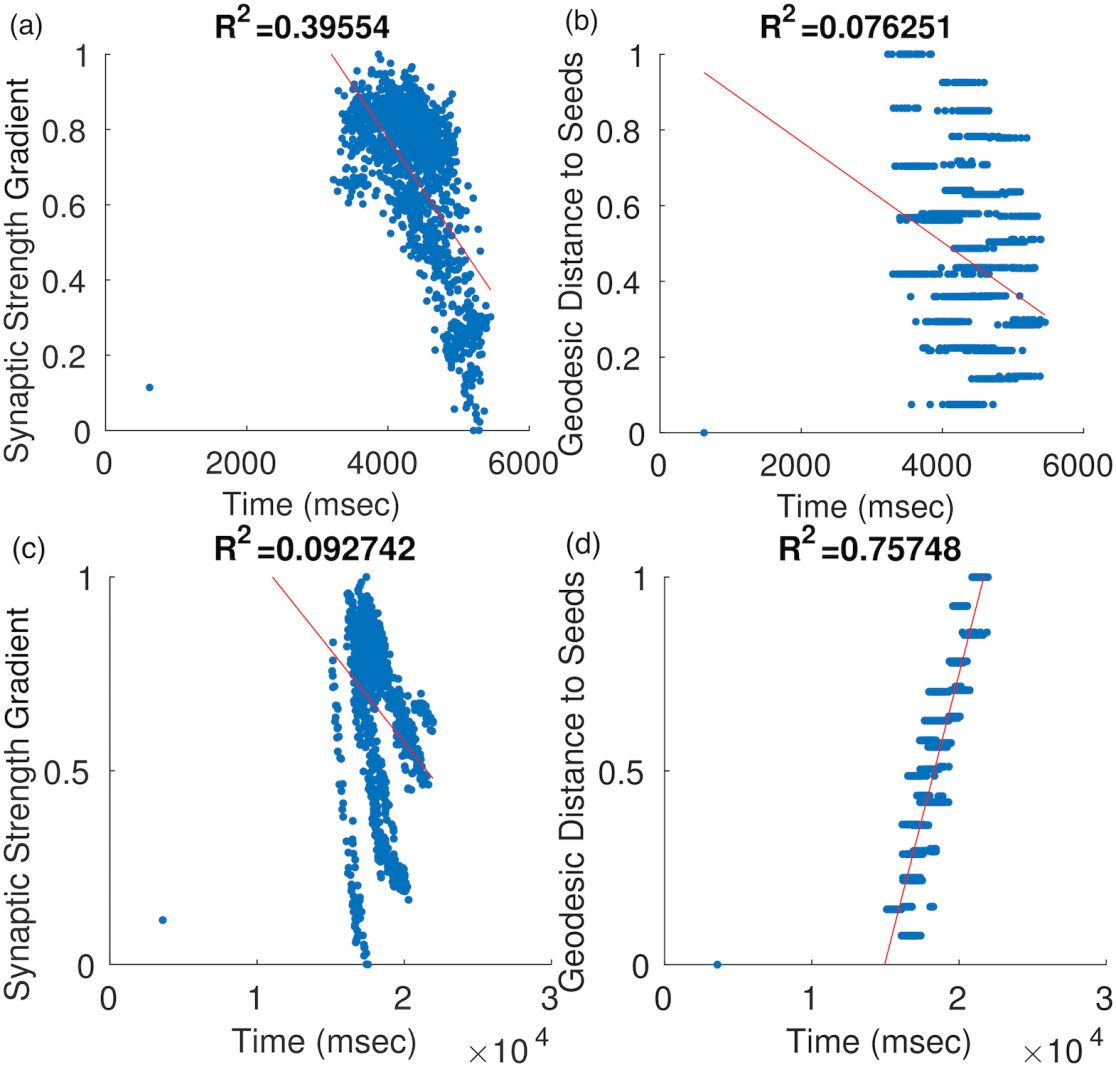
Each point indicates a neuron *i*, at the time of its cellular death *t*_*i*_ and its respective *SSG*_*i*_ (left) or *GDS*_*i*_ (right) value for simulations primarily driven by passive diffusion of pathogenic protein (seed L2FS, low misfolding rate, strong active transport, no synaptic transfer and no spread selectivity). (a) SSG and (b) GDS with soluble pathogenic protein. (c) SSG and (d) GDS with insoluble pathogenic protein.

In order to assess the vulnerability of specific neurons we calculated for each neuron its Geodesic Distance to the Seed (*GDS*) and its “Synaptic Strength Gradient” (*SSG*, Eq 18), which is the difference between the sum of its presynaptic connection strengths and the sum of its postsynaptic connection strengths. Neurons with a high SSG have many and strong presynaptic connections, but few and weak postsynaptic connections (here termed ‘bottleneck’ neurons). Modulation of information transfer between input and output connections is a basic concept of neuron biology and neurons have widely varying input:output relations [27]. We hypothesised that neurons with a high SSG or a low GDS value are more vulnerable and reach cellular death earlier. In Fig 4, every neuron is plotted as a point, using their respective SSG and GDS values against the simulation time that they reached cellular death; we performed linear regression and calculated *R*^2^ values in order to assess the relation between these neuronal characteristics and time to cellular death. We will refer to SSG *R*^2^ as the ‘bottleneck’ neuron survival characteristic and to GDS *R*^2^ as the distance to seed survival characteristic.

We quantified the asymmetry of network damage *ASY* by taking the maximum value over time of the standard deviation of the neuronal toxicity (Eq 22). We assume that a high variation in neural network damage is similar to what one would call asymmetric neural network damage. This maximum value typically occurred during the first few cellular deaths. We also calculated the time it took for all neurons to reach cellular death (time to network breakdown—TTNB) in each simulation. Table 1 indicates the magnitude of the effect each parameter had on SSG *R*^2^, GDS *R*^2^, ASY and TTNB.

**Table 1:** Impact of simulation parameters on ‘bottleneck’ neuron survival characteristic (SSG *R*^2^), distance to seed survival characteristic (GDS *R*^2^), spread asymmetry (ASY) and time to network breakdown (TTNB). Number of +/- signs indicate strength of impact on the metric compared to a baseline observation, with 0 indicating no impact. ^1^ N/A since initial protein seed concentrations were different (see Materials and methods; for similar seeds, TTNB would be much higher with soluble pathogenic protein). ^2^ The first three seeds had higher values. ^3^ Layer 4 and 5 seeds had lower values, whereas layer 6 had highest values. ^4^ *,* indicates the relationships: [tendency of pathogenic protein to avoid intercolumnar connections vs no spread selectivity], [tendency of pathogenic protein to spread via intercolumnar connections vs no spread selectivity].

Parameter	SSG *R*^2^	GDS *R*^2^	ASY	TTNB
1) Network connectivity	0	0	0	0
2) Protein insolubility	−−	++	++	N/A^1^
3) Seed location	^2^	^3^	0	0
4) Misfolding rate	0	0	+	−
5) Diffusion speed	+++	+++	++	0
6) Active transport strength	++	+	++	−−
7) Synaptic transfer strength	++	+	++	+
8) Spread selectivity^4^	+++, +	−−, −−	+, +++	0, 0

In order to assess each parameter’s impact on protein spread patterns, we quantified the similarity and time to convergence between the order of cellular deaths for all pairs of simulations. Given a pair of simulations *s* and $\bar{s}$, we created the sets $G_{n}^{s},G_{n}^{\bar{s}},n\in \left\{1,...,N\right\}$ (*N* is total number of neurons), indicating the set of the first *n* neurons to reach cellular death in simulation *s*, $\bar{s}$. We calculated the Dice coefficient (a measure of similarity between two sets) $D_{s,\bar{s}}\left(n\right)$ (Eq 19) between sets $G_{n}^{s}$, $G_{n}^{\bar{s}}$ for *n* ∈ {1, …, *N*}. The higher the Dice coefficient value between two sets, the larger the number of dead neurons in common between the sets. We defined time to convergence as the time $t_{c}\left(s,\bar{s}\right)$ (Eq 20), normalised by *N*, that the Dice coefficient reached and continued to exceed a high threshold value *t*_*conv*_ = 0.8. After convergence, since the Dice coefficient remains high, the two simulations show similar spread patterns. Simulations with a low $t_{c}\left(s,\bar{s}\right)$ (*i.e.* early convergence) have similar spread patterns during the entire simulation.

**Fig 5:**
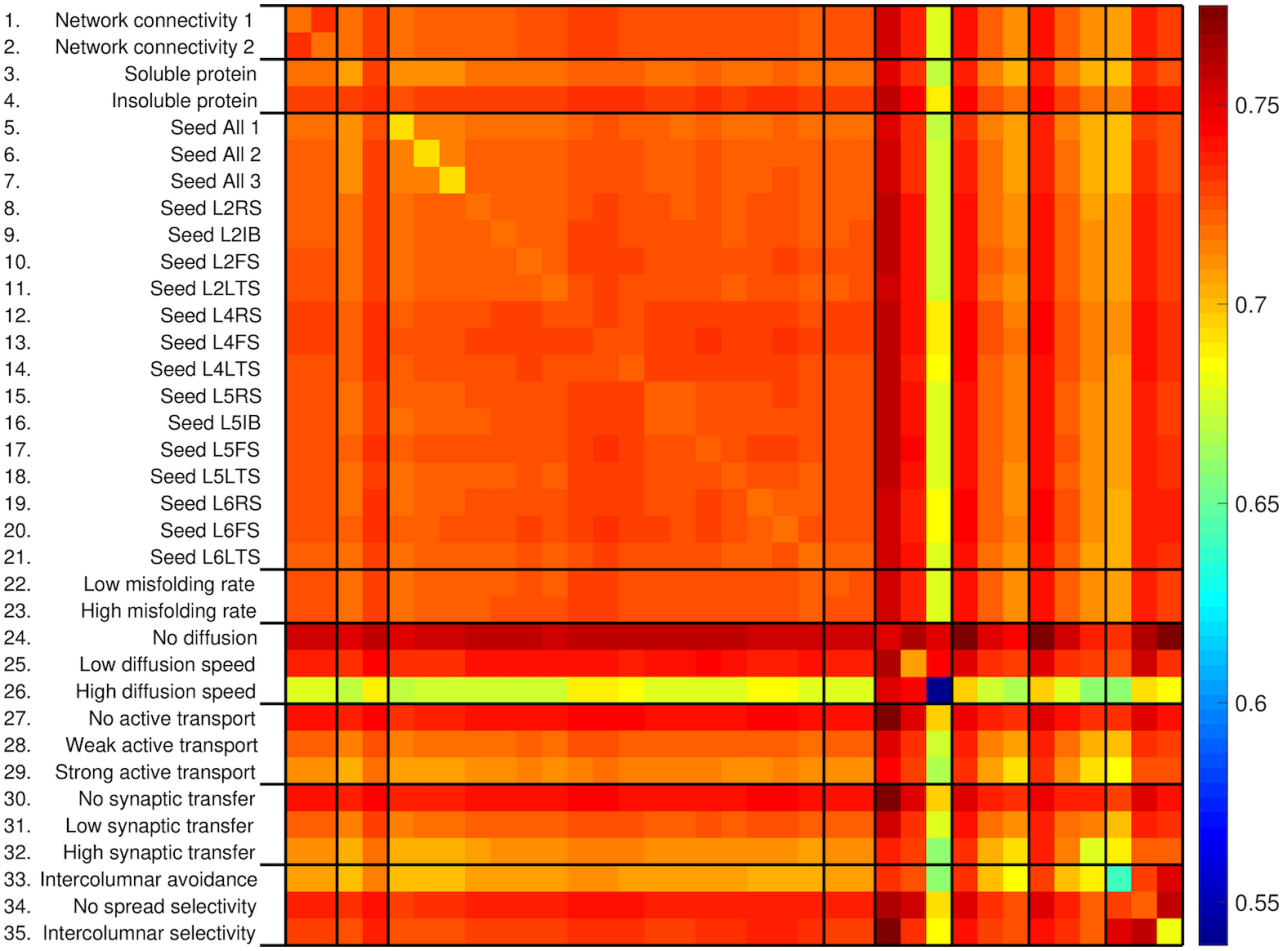
Heatmap of mean time to convergence between all pairs of simulation sets *H*_*i*_ (*e.g.* a value of 0.65 indicates that convergence occurred after 65% of neurons reached cellular death). The x-axis labels are replicated from the y-axis labels.

Fig 5 summarises the time to convergence for all pairs of simulations. We created the sets *H*_*i*_, *i* ∈ {1, …, 35}, each of which includes all simulations with one common parameter value indicated by the underscript *i*. The value of *i* is indicated on the *i*-th row of the y-axis of Fig 5. For example, all simulations with a ‘Low misfolding rate’ belong to set *H*_22_. We computed a matrix $\text{CONV}\in R^{35\times 35}$, where element **CONV**_*ij*_ is the mean time to convergence between all simulations in set *H*_*i*_ against all simulations in set *H*_*j*_ (Eq 21). Comparing diagonal elements illustrates the effect of a single protein mechanism on time to convergence, whereas by comparing non-diagonal elements within a row or a column one can study how variation of a second protein mechanism affects time to convergence.

**Fig 6:**
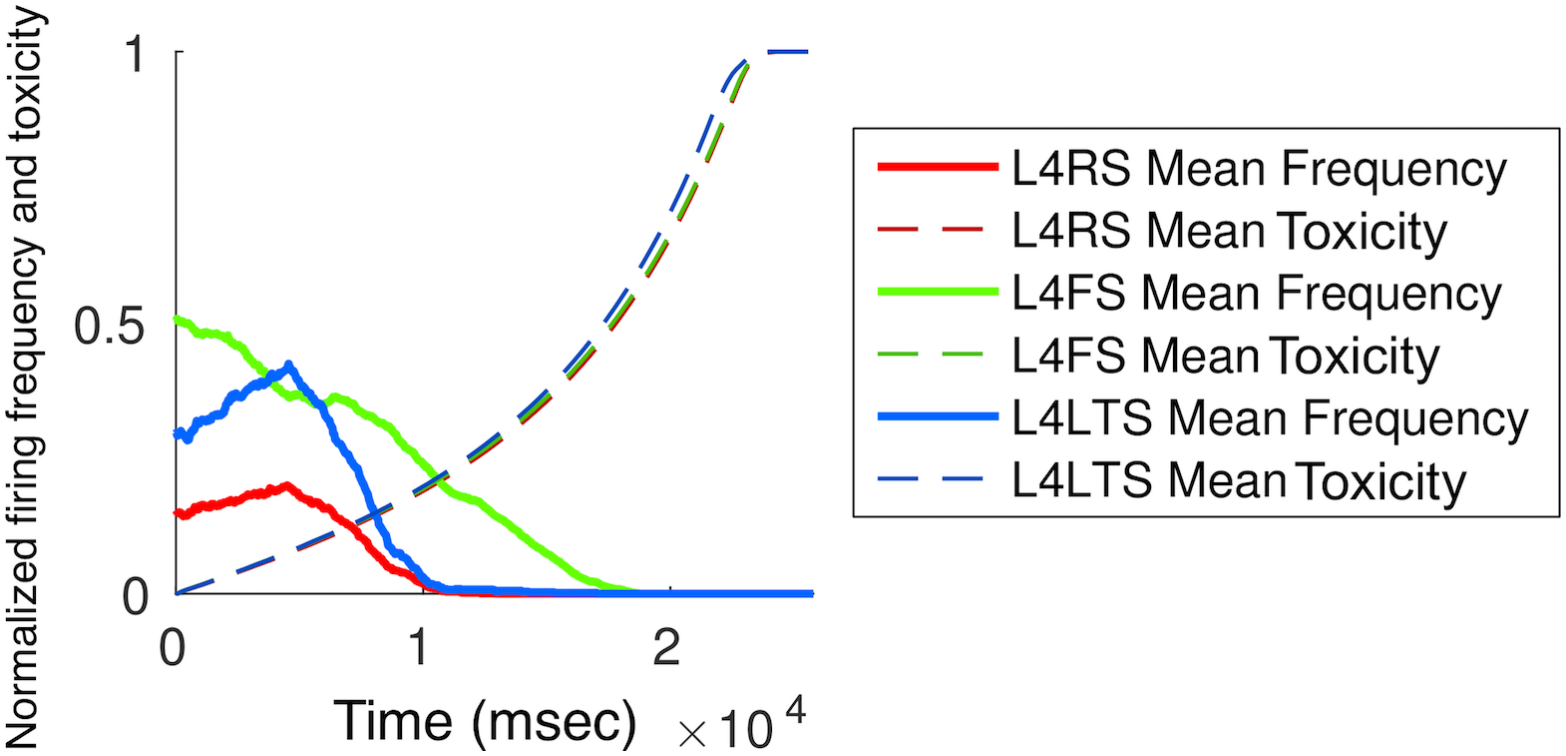
Mean toxicity and firing frequencies over time for layer 4 neurons of the first cortical column, grouped by neuronal type.

Fig 6 displays the mean firing frequencies and toxicity over time for certain neuron types in a simulation with an increase of firing voltage thresholds toxic effect.

## Discussion

Across simulations, passive diffusion was the primary driver of the spread patterns (Fig 5), strongly influencing neuronal survival characteristics based on distance to seed (GDS) and synaptic input:output relations (SSG) as well as the asymmetry of network breakdown (Fig 4, Table 1); in combination with soluble protein, spread patterns showed a relationship with the input:output (SSG) metric, whereas with insoluble protein spread patterns showed a relationship with the distance to seed (GDS) metric. Assuming a primarily anterograde spread of protein, our findings suggest that a high SSG value (high neuronal input:output) may confer vulnerability to the neurodegenerative process, rather than general connectedness (high neuronal input+output), which is typically considered [4] a determinant of neuronal survival. This accords with recent formulations assigning the diffusive spread of pathogenic proteins a central role in the evolution of neurodegenerative proteinopathies [1–5, 15] and with evidence from animal models of protein propagation [6].

Increased misfolding rate and active transport of pathogenic protein hastened network breakdown, consistent with an amplification of intracellular toxic effects, as predicted empirically [5, 6, 28]. A higher misfolding rate hastened neurodegeneration (reduced TTNB) far more than a higher initial seed concentration in the case of insoluble pathogenic protein, in agreement with in vivo data [22].

The asymmetry of network breakdown increased with insoluble protein, increased misfolding, diffusion speed, active transport, synaptic transfer or when protein had the tendency to spread selectively via intercolumnar connections: these mechanisms tend to ‘focus’ neuronal toxicity in particular neurons, a mechanism previously proposed to underpin the strikingly asymmetric atrophy profiles of TDP-43-opathies [3]. In addition, inspection of Fig 5 suggests that the conjunction of particular protein and network factors promoted more rapid convergence of patterns of network breakdown, in keeping with a molecular nexopathy mechanism [3]. Pathogenic protein solubility, higher diffusion speed, stronger active transport and synaptic transfer and any spread selectivity all consistently accelerated convergence of the spread pattern.

Our findings endorse an important role of local neuronal geometry in modulating network breakdown (Figs 4, 5 and 6, Table 1). Whereas changing the overall network connectivity had no effect, the laminar location of seeded neurons importantly affected the pathogenic protein spread pattern. This might suggest a computational basis for the exquisite histopathological selectivity and regional vulnerability that characterise a range of neurodegenerative diseases [1–6, 15, 23]. Although our simulations were not designed primarily to capture alterations in neuronal electrophysiology, the relatively simple model parameters yielded complex neuronal activity profiles that showed a dependence both on neuronal type and time. For example, L4LTS neurons showed an initial increase in firing frequency under a ‘loss of function’ toxic effect (Fig 6), potentially due to the removal of inhibitory effects from connected neurons and in line with previous predictions based on biological disease models [23].

Especially pertinently, our simulations identify factors that might constitute targets for therapeutic manipulation. For example, increased diffusion speed in the context of a soluble pathogenic protein tended to prolong overall network survival. This follows as faster diffusion spreads protein more evenly in the network, promoting overall clearance of soluble pathogenic protein: a potential therapeutic mechanism that has attracted much recent interest [19, 20, 28]. Similar considerations may apply to synaptic transfer of pathogenic protein, which was also protective (Table 1) and has also been proposed as a target for future interventions [14]. In Alzheimer’s disease, amyloid-beta is associated with toxic hyperexcitability [16, 29] (which in turn leads to even more pathogenic protein to be released from affected neurons). Our synaptic transfer mechanism was protective for individual neurons with a high firing frequency, but in combination with the hyperexcitability toxic gain effect, it was protective on the entire network-scale (increase of TTNB, Table 1) for both soluble and insoluble pathogenic protein. Alzheimer’s disease is the result of a complex interplay between amyloid-beta and tau proteins: the neuronal loss caused by tau coupled with the neuronal hyperexcitability caused by amyloid beta could have the effect of spreading tau outside zones of amyloid deposition, leading to differential tissue distributions of the two proteins, in line with recent cellular and neuroimaging data [29, 30].

## Conclusion

Here we have shown that modelled pathogenic protein mechanisms and network properties drive patterns of network breakdown in a simulated cortical neural network. Crucially, modification of protein and network parameters produced consistent and convergent patterns of protein spread, rather than random sequences of cellular deaths.

The potential of computational modelling techniques to simulate neural network disintegration remains largely unexplored. Such techniques seem ideally suited to define the ‘structural logic that governs the biological effects’ of neurodegenerative pathologies [22]. Our findings suggest that a small artificial neural network under a handful of relevant parameters can generate diverse, biologically plausible behaviour that is broadly relevant to human neurodegenerative diseases and consistent with empirical data.

A major limitation of all such modelling approaches is the need to simplify (sometimes radically) in order to capture a few mechanisms of pathogenic proteins which are likely to be of general relevance. There are a number of ways in which our model should be refined in future work. Some important factors that we have not addressed here, but which are likely to contribute to the pathogenesis of neurodegenerative diseases, include chaperone proteins, glial cell interactions with neurons, protein aggregates of different sizes and the recruitment of additional proteins by larger aggregates, protein spread via the extracellular compartment and the operation of intracellular endosomes. Each of these could potentially be assessed in developing our model further and making it a more realistic simulation of the complexity of actual neurodegenerative diseases. However, we believe that even in the simplified approach presented here, computational modelling approaches show promise in assisting in the development of future diagnostic tools. If such approaches identify candidate properties of pathogenic proteins that drive neural network breakdown, then this in principle would allow culprit proteins to be inferred from particular profiles of network breakdown that are observed empirically (for example, using brain imaging).

Further work is required to test the model against a range of diseases and data derived in vitro and from animal models. By fitting the model to such data, one can derive which specific parameters and models are likely to govern each individual pathogenic protein. As computational models are continually refined based on new neurobiological findings (*e.g.* production of protein per neuron per unit of time), this framework could quickly and easily test a variety of hypotheses regarding pathogenic proteins, so that further neurobiological research can focus on hypotheses which were found to be more likely to be true. The ultimate goal will be to determine how computational models of micro-circuits scale to whole-brain anatomical profiles of human disease, at which scale neuroimaging can be used as validation to learn which parameters and models represent each neurodegenerative disease. This framework will potentially be able to predict neurodegenerative disease progression, based on protein and neural network characteristics and assess the impact of candidate modulatory factors, with clear implications for rational drug discovery.
